# Pre-clinical training, technical adjustment and human case experience of transoral robotic surgery using Versius System^☆^

**DOI:** 10.1016/j.bjorl.2024.101515

**Published:** 2024-10-04

**Authors:** Guilherme Reimann Agne, Gustavo Nunes Bento, Marcelo Belli, Gustavo Becker Pereira, Renan Bezerra Lira, Leandro Luongo Matos, Luiz Paulo Kowalski

**Affiliations:** a Cirurgia de Cabeça e Pescoço no Grupo PESCOP, Balneário Camboriú, SC, Brazil; b CMRSurgical Ltd,Coloproctologia, Balneário Camboriú, SC, Brazil; c A.C. Camargo Cancer Center, Departamento de Otorrinolaringologia e Cirurgia de Cabeça e Pescoço, São Paulo, SP, Brazil; d Hospital Albert Einstein, Programa de Cirurgia Robótica, São Paulo, SP, Brazil; e Universidade de São Paulo, Faculdade de Medicina, Departamento de Cirurgia de Cabeça e Pescoço, São Paulo, SP, Brazil; f Hospital Albert Einstein, Cirurgia, São Paulo, SP, Brazil

**Keywords:** Robotic surgical procedures, Surgical robotics, Robotics computer-assisted, Head and neck neoplasms, Head and neck surgery

## Abstract

**Objective:**

This study describes the training experience with the Versius Robotic System (CMR Surgical) for Transoral Robotic Surgery (TORS), the setup of the robot and its applicability in a human case.

**Methods:**

A model mannequin with anatomical characteristics simulating the TORS working space was used for training purposes. Optimal docking, positioning of robotic arms and placement of instruments were defined using the mannequin. A human patient was selected based on the inclusion criteria, and a standardized protocol for robotic techniques was developed. The surgical procedure was conducted in accordance with ethical guidelines.

**Results:**

The synthetic polymer mannequin mimicking human tissues and anatomy was used to define the robot set up for TORS. The patient was indicated for bilateral tonsillectomy due to recurrent episodes of tonsillitis. Bipolar Maryland and monopolar scissors were used. The procedure was uneventful. The patient remained hospitalized for 1 day and returned to work activities within 6-days, with no complications.

**Conclusion:**

The specialized mannequin proved to be effective in the simulation, training proposal and set up standardization. Furthermore, the Versius robot was found to be safe and achieved excellent results during surgery. Despite being very promising, the Versius system is still in its developmental phase and requires larger-scale samples for a more comprehensive evaluation.

**Level of evidence:**

4.

## Highlights

• Transoral Robotic Surgery is well-established using the DaVinci system.
• Transoral robotic surgery with the Versius System is currently in its initial stage.
• A mannequin model was effective for training and setup configuration.
• The first human case demonstrated feasibility and excellent results.
• In our preliminary experience, Versius proved to be a promising robotic system.

## Introduction

Transoral surgeries present visibility- and handling-related limitations due to restricted access.^1^ Robotic surgery is used in head and neck procedures due to the need to penetrate deeper regions within the oral cavity, where the visibility and manual reach of surgeons may be constrained.^2^ Transoral Robotic Surgery (TORS) has emerged as a promising option for the treatment of head and neck tumors, as it allows the use of articulated instruments and high-quality 3D vision, thus making it a viable and safe surgical option deeper in the oral cavity.^3^ Furthermore, this approach contributes to reducing or eliminating the need for radiotherapy among patients with oropharyngeal squamous cell carcinomas; furthermore, TORS aims to alleviate potential sequelae resulting from such treatment.^4^, ^5^, ^6^, ^7^

The main aspect of TORS is the resection of tumors of the tonsil and base of the tongue, which can be performed via open surgery; this procedure has an important functional and aesthetic impact, as the use of radiotherapy is associated with several subsequent adverse effects.^8^, ^9^, ^10^, ^11^ TORS also has expanded indications for surgery of the hypopharynx, parapharynx, and larynx.^12^ Studies have reported good outcomes from the use of robots in the surgical treatment of obstructive sleep apnea in selected patients, suggesting that robots are a promising alternative to continuous positive airway pressure therapy.^13^, ^14^, ^15^ Furthermore, in head and neck surgery, TORS can be used for approaches that have aesthetic aims, such as retroauricular neck dissection and robotic transvestibular thyroidectomy.^16^, ^17^

The progression of robotic surgery in the head and neck followed the development of Da Vinci’s platform (Intuitive Surgical Inc., Sunnyvale, CA, USA). Nevertheless, the expiration of Intuitive's patents on the robotic system paved the way for technological advancements by other companies in the market. Additionally, alternative robotic systems may prove more available due to their lower costs.^18^, ^19^

The Versius robotic system, which was developed by CMR Surgical (Cambridge, United Kingdom), has yielded comparable outcomes to those of other robots across various specialties. Notably, this robot stands out due to its uncomplicated docking system, facilitated by individual mobile units (i.e., bedside units). The robot arms are equipped with 6-mm instruments with a 720 ° rotation capacity.^19^, ^20^, ^21^

In contrast to da Vinci’s robotic system, the Versius System needs to define a pivot point along the instrument in each arm; in this location, the instrument remains stable and does not move. Typically, the pivot point is established on the abdominal wall using a trocar – this process is referred to as port training. The challenge in head and neck surgery is the absence of a natural pivot point, such as the abdominal wall, and the need to perform virtual port training manually at some point along the path.^19^

In 2021, a preclinical study utilizing cadavers demonstrated the feasibility of performing TORS and elucidated the limitations of the Versius robot.^22^ In 2024, the same author published the first series of human cases.^23^ However, different approaches, settings, and interpretations of the CMR Versius platform were noted. This study aims to offer alternative options and fresh perspectives for TORS by examining the use of the Versius System on a mannequin and providing an accurate description of the technique. Subsequently, our team of experts conducted a real-life experiment. We consider this type of research promising and necessary in today's robotic surgery landscape, aligning with previous studies and highlighting the importance of continuous improvement in the field.

## Methods

### Survey design

This study was conducted in accordance with the Declaration of Helsinki and was approved by the Institutional Review Board (IRB number 6.502.205). The participants received a comprehensive explanation of the potential risks associated with the procedure and subsequently signed the informed consent form before being enrolled. The authors affirm that the participants provided informed consent for publication of the images.

Initially, a mannequin model was created by ProDelphus™, which features anatomical characteristics that simulate the TORS operating field. Moreover, the mannequin exhibits prominent tonsils and a lesion on the base of the tongue to simulate resections.

Through the utilization of the mannequin, the optimal docking and positioning of the robotic arms, as well as instrument placement, were defined. After the technical definition and training facilitated by the mannequin, cases were selected for the initial human procedures. The inclusion criteria for these transoral procedures were as follows: patients aged 18 years and older; patients with benign diseases of the oropharynx for which surgical treatment was indicated; and patients for whom the use of a robotic system was optional, thus allowing easy conversion to a conventional operation without a robot and ensuring patient safety.

For this study, the robotic system and technique used were standardized to develop protocols that could support similar or more extensive surgeries. Due to the descriptive nature of this study, the results were divided into mandatory procedures and training, followed by a detailed description of the surgical case in a human patient.

## Results

### Training

**Fig. 1:**
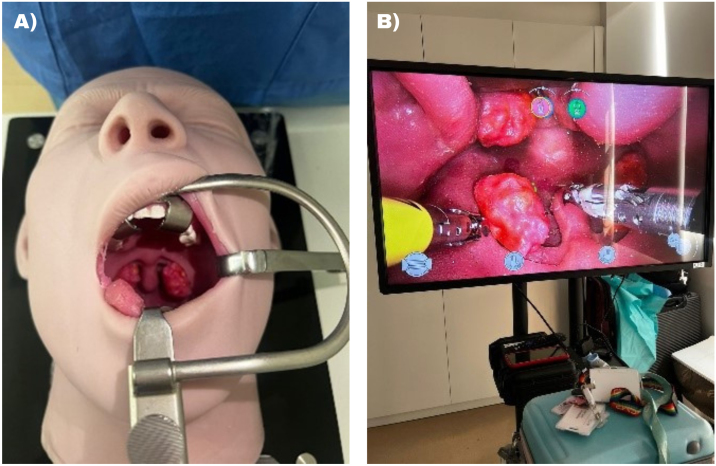
(A) Mannequin positioned with the Davis-Boyle retractor. (B) Camera view of the mannequin's oral cavity with the robot's instruments.

A specialized prototype was created to simulate the human head, encompassing both the oral cavity and pharynx (Fig. 1). We employed a robotic training system for preclinical docking and port training programming.

Various configurations were tested until an optimal setup was determined for the mannequin. The objective was to ascertain a robot position that would allow adequate exposure and instrument stability while minimizing collisions. Measurements were recorded and replicated in the first case. The training sessions on the mannequin have been shown to be safe and feasible for proceeding to the clinical phase.

### Patient positioning

The patient was placed in the surgical bed with his head away from the anesthesia cart, optimizing the space for the Versius surgical units. A slight cervical extension was obtained with a back cushion placed at the level of the scapula. This cervical extension appears to enhance the approach angle of the robotic instruments. A Davis-Boyle mouth gag was then employed, providing adequate exposure.

### Docking

**Fig. 2:**
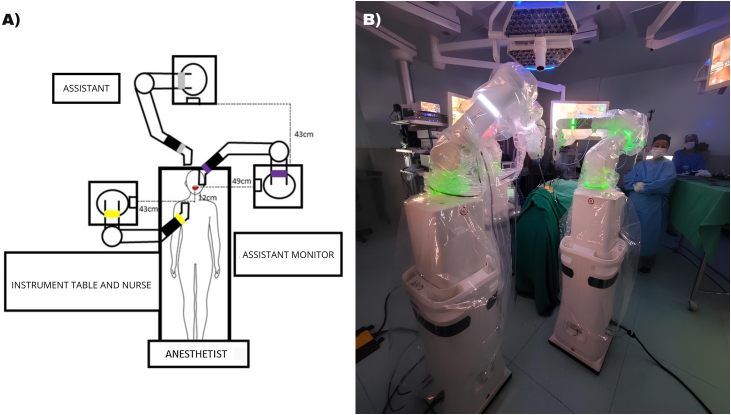
(A) Organizational layout of the operating room and positioning of the robot (B) Operating room arranged as described.

The right robotic bedside was docked laterally 43 cm away from the maxillary central incisors. The center of the bedside was 12 cm caudal to the transverse plane of the maxillary incisor teeth. The “C” arm was positioned with the concavity facing cranially, with the arm’s height set to 13 cm (Fig. 2).

The left bedside was docked 49 cm away from the maxillary central incisors, with the center of the bedside in the transverse plane of the maxillary incisors. The “C” arm positioning was performed with the concavity facing caudally, with the height set at 12.5 cm. The bedside endoscopic camera was docked in the midline above the patient's head 43 cm above the vertical plane of the left arm’s bedside edge and arranged in the “Z” position. This allowed for the positioning of an assistant on both sides of the headboard.

### Port training

**Fig. 3:**
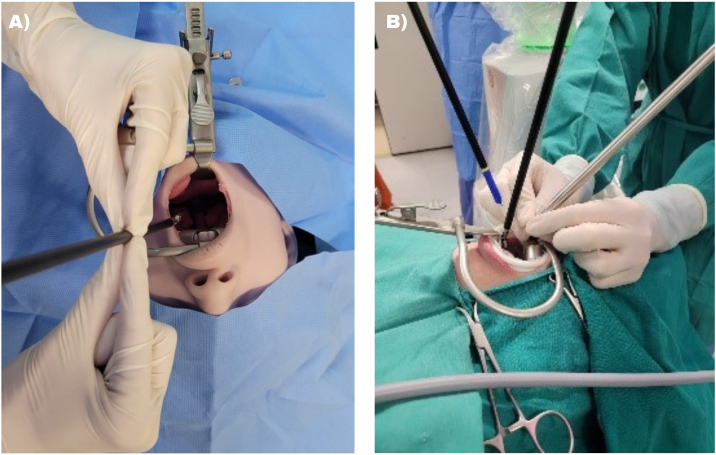
(A) Port training for the right arm performed at a point 15 cm above the labial commissure and directed towards the uvula during training with the mannequin. (B) Port training for the endoscopic camera being conducted 2 cm above the central incisors during surgery.

Initially, the instrument to be used is positioned with the tip close to the desired destination (using the uvula as a reference) and passing through the desired pivot point 15 cm away from the ipsilateral labial commissure. A 3- to 5-cm indentation is made to make it possible to insert after port training. Port training must be conducted with a surgeon and an assistant, with one responsible for measurements and pivot positioning of the port training while both hands perform a pincer movement (Fig. 3A), followed by another responsible for performing the arm movement to register the port training. Bipolar Maryland and monopolar scissors were used in our experiment. The camera chosen was 30 ° facing downward, and port training was carried out 2 cm in height from the central incisor aiming at the uvula (Fig. 3B).

### Clinical case

A 37-year-old female presented with six recurrent episodes of tonsillitis in the last 12 months, indicating the need for bilateral tonsillectomy. Tonsillectomy was selected as the first surgical procedure due to its ability to be easily followed up with conventional surgery, if necessary. Robot docking and port training were conducted in accordance with previous descriptions. Maryland bipolar forceps were used on the ipsilateral side of the tonsil to be operated on to optimize space and reduce collisions. With these forceps, inferior and medial traction of the tonsil was performed. Monopolar scissors were used in the other arm, crossing with Maryland.

**Fig. 4:**
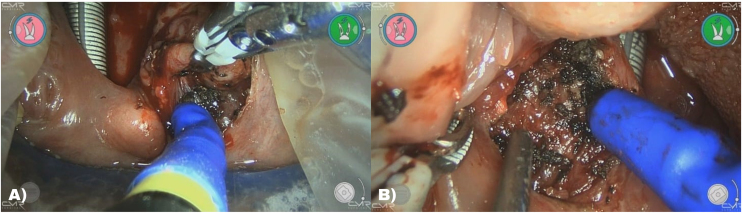
(A) Arrangement of the instruments in right tonsillectomy. Note the inferior and medial traction performed crossing the Maryland. (B) Surgical bed after removal of the left palatine tonsil.

The approach began with medial traction of the tonsil and the use of monopolar scissors with cut energy to the mucosa at the upper pole of the tonsil until the identification of the capsule. Subsequently, the scissors were used in coagulation mode, and the procedure continued with tonsil capsular detachment. The assistant used a suction instrument to apply countertraction and keep the field clean; occasionally, a malleable retractor was employed. Bleeding in the surgical field was minimal and controlled with monopolar energy. After removing the specimen and exposing the surgical bed, hemostasis was achieved with bipolar energy (Fig. 4).

A previous study conducted with cadavers suggested the use of the Alexis® as a wound protector and instrument support, we used the Marlex® wound protector for right tonsillectomy and removed it for left tonsillectomy for comparison purposes.^22^ We did not encounter any issues with stability or instrument tremors, and having a wound protector adds costs without clear advantages.

The positioning time (docking and port training) was 35 min. The console time for the right tonsilla was 48 min. A new 10-minute positioning adjustment was made to perform the contralateral resection. The console time for the left amygdala was 32 min. The patient remained hospitalized for one day and returned to work activities within 6 days. The patient's pain was managed with the use of simple analgesics, anti-inflammatory drugs, and occasionally codeine.

## Discussion

The preclinical study involving the mannequin for robotic procedures using the Versius platform represents a significant advancement in simulating head and neck surgeries on real patients, highlighting the efficacy and practicality of this approach. The resemblance between the mannequin and the clinical scenario (Fig. 1B and Fig. 4A) underscores the importance of these models and their ability to accurately replicate human anatomical features. This methodology not only allows surgeons to develop crucial skills but also facilitates their acclimation to the use of robotic instruments. The quality of synthetic mannequins mimicking human tissues must be highlighted. In terms of standardization and optimization of the surgical procedure, the observed equipment configurations proved to be effective and safe.

In the preclinical study by Faulkner et al., the Alexis® retractor (Ethicon™) was used to better stabilize the instruments.^22^ In our experience, despite offering interesting thermal protection to the mucosa and lip, the use of a wound protector did not affect the stability of the instruments. One possible explanation for this discrepancy could lie in the definition of surgical port training. In our study, we observed that a 5-cm adjustment in the surgical port training definition, moving it to a more distant position, resulted in improved instrument stability. This adjustment may have influenced the mechanics of the surgery, thus providing a more favorable configuration that reduced instrument vibration or wobbles, as reported by Faulkner et al.^22^

In a recent publication, Faulkner et al. reported the first series of cases in which TORS with the Versius system was used. In this study, 15 patients with benign disease and 15 with cancer were included. Differences in the configurations and techniques employed were observed compared to our study. Initially, in our experience, docking was more effective when the distance from the reference point was greater than the study mentioned above.^22^ Another difference lies in the more distant positioning of the training port pivot point, as mentioned earlier. Additionally, there was a discrepancy in the selection of instruments used. While Faulkner and his colleagues opted for the hook associated with bipolar Maryland, our team found the use of monopolar scissors in conjunction with bipolar Maryland to be satisfactory. Finally, the strategy adopted by the study to incorporate a fourth arm was deemed interesting, but its efficacy needs to be evaluated separately due to space limitations.^23^

The main technical challenge encountered during the procedure was collisions between surgical instruments and cameras due to working space limitations. This is a common issue in other robotic systems and emphasizes the importance of adequate exposure of the oral cavity and retractor positioning in TORS. In the described case, the small oral cavity of the patient contributed to an increased frequency of collisions.

Collisions were observed at the onset of the surgery, and adjustments in the positioning of the robot's arms and the configurations of its instruments were needed. These fine-tuned adjustments ensure that the robot maintains proper positioning throughout the surgical procedure, thus preventing potential interference. The main improvement was in the use of inferior and medial traction with the ipsilateral hand crossing the instruments (Fig. 4A). This maneuver allowed the procedure to continue without collisions.

One significant difficulty observed was the lack of available bipolar grasper forceps for use with the Versius system, as there is only one available grasper without options for energy application. In the described case, traction on the palatine tonsil was applied using a Maryland instrument; however, while this instrument was delicate, it was not specifically designed to distribute pressure adequately on a fragile structure. The availability of a wider variety of surgical instruments compatible with the Versius system is crucial to optimize the effectiveness and safety of procedures performed with this robotic system. In addition, ultrasonic sealing devices are already being used in some countries but are not yet approved for use in Brazil by the regulatory agency.

The 6-mm instruments and greater rotation capability compared to Da Vinci (720 ° vs. 540 ° present promising prospects for the Versius robot. Despite showing excellent results compared to those of other robots, the Versius Robotic System still needs alignment in terms of technical standardization. Technological standardization is crucial for minimizing collisions, reducing surgical time and progressing to more complex surgeries. Additionally, in terms of surgical time, one must consider the learning curve, which reflects greater long-term performance and efficiency.

The main limitation of our study is inherent to case reports such as this one. Therefore, it is imperative to conduct additional studies for technical development and to increase experience. Anatomically complex regions, such as the base of the tongue and the supraglottic larynx, which may present additional challenges during surgery, need to be investigated regarding the safety and efficacy of Versius use. We recommend that further head and neck surgeries with Versius should be performed following a research protocol with adequate inclusion criteria until the technical limits of the robot are better defined. This approach will facilitate a comprehensive analysis of outcomes and provide a more nuanced understanding of the system's capabilities and constraints across diverse clinical scenarios. The use of surgical robots, such as the Versius system, represents a significant advancement in surgical practices, offering improved visualization and precise maneuvers in limited anatomical spaces. This advancement contributes to safer and more effective procedures, especially in complex anatomical regions such as the head and neck.

## Conclusion

Despite the widespread adoption and well-established reputation of da Vinci’s system, Versius has emerged as a promising alternative, and our preliminary experience demonstrated its efficiency and safety. Furthermore, the developed specialized mannequin proved to be effective in the simulation and training. Finally, it is important to acknowledge that the Versius system is still in its developmental phase and requires larger-scale samples for a more comprehensive evaluation.

## Financial support

This study has no financial relationship with CRM Surgical or any other companies. The costs associated with the use of the robotic system were subsidized by Unimed Hospital.

## Conflicts of interest

The author Gustavo Becker is an official medical proctor for CMR Surgical. Others authors have no relevant financial or non-financial interests to disclose

## References

[1] Hockstein N.G., O’Malley B.W., Weinstein G.S. (2006). Assessment of intraoperative safety in transoral robotic surgery. Laryngoscope..

[2] Tamaki A., Rocco J.W., Ozer E. (2020). The future of robotic surgery in otolaryngology – head and neck surgery. Oral Oncol..

[3] Poon H., Li C., Gao W., Ren H., Lim C.M. (2018). Evolution of robotic systems for transoral head and neck surgery. Oral Oncol..

[4] Hanna J., Morse E., Brauer P.R., Judson B., Mehra S. (2019). Positive margin rates and predictors in transoral robotic surgery after federal approval: a national quality study. Head Neck..

[5] de Almeida J.R., Li R., Magnuson J.S., Smith R.V., Moore E., Lawson G. (2015). Oncologic outcomes after transoral robotic surgery: a multi-institutional study. JAMA Otolaryngol Head Neck Surg..

[6] Chang E.H.E., Kim H.Y., Koh Y.W., Chung W.Y. (2017). Overview of robotic thyroidectomy. Gland Surg..

[7] Douglas J.E., Wen C.Z., Rassekh C.H. (2020). Robotic management of salivary glands. Otolaryngol Clin North Am..

[8] Miles B.A., Posner M.R., Gupta V., Teng M.S., Bakst R.L., Yao M. (2021). De-escalated adjuvant therapy after transoral robotic surgery for human papillomavirus-related oropharyngeal carcinoma: the sinai robotic surgery (sirs) trial. Oncologist..

[9] Scholfield D.W., Gujral D.M., Awad Z. (2020). Transoral robotic surgery for oropharyngeal squamous cell carcinoma: improving function while maintaining oncologic outcome. Otolaryngol Head Neck Surg..

[10] Nichols A.C., Theurer J., Prisman E., Read N., Berthelet E., Tran E. (2019). Radiotherapy versus transoral robotic surgery and neck dissection for oropharyngeal squamous cell carcinoma (ORATOR): an open-label, phase 2, randomised trial. Lancet Oncol..

[11] Nichols A.C., Theurer J., Prisman E., Read N., Berthelet E., Tran E. (2022). Randomized trial of radiotherapy versus transoral robotic surgery for oropharyngeal squamous cell carcinoma: long-term results of the ORATOR Trial. J Clin Oncol..

[12] Mella M.H., Chabrillac E., Dupret-Bories A., Mirallie M., Vergez S. (2023). Transoral robotic surgery for head and neck cancer: advances and residual knowledge gaps. J Clin Med..

[13] Oberhelman N., Bruening J., Jackson R.S., Van Abel K.M., Sumer B., Holsinger F.C. (2024). Comparison of da vinci single port vs si systems for transoral robotic-assisted surgery: a review with technical insights. JAMA Otolaryngol Head Neck Surg..

[14] Lin H.S., Rowley J.A., Folbe A.J., Yoo G.H., Badr M.S., Chen W. (2015). Transoral robotic surgery for treatment of obstructive sleep apnea: factors predicting surgical response. Laryngoscope..

[15] Meccariello G., Cammaroto G., Montevecchi F., Hoff P.T., Spector M.E., Negm H. (2017). Transoral robotic surgery for the management of obstructive sleep apnea: a systematic review and meta-analysis. Eur Arch Otorhinolaryngol..

[16] Miller S.C., Nguyen S.A., Ong A.A., Gillespie M.B. (2017). Transoral robotic base of tongue reduction for obstructive sleep apnea: a systematic review and meta-analysis. Laryngoscope..

[17] Lira R.B., Chulam T.C., Kowalski L.P. (2017). Safe implementation of retroauricular robotic and endoscopic neck surgery in South America. Gland Surg..

[18] Weinstein G.S., O’Malley B.W., Magnuson J.S., Carroll W.R., Olsen K.D., Daio L. (2012). Transoral robotic surgery: a multicenter study to assess feasibility, safety, and surgical margins. Laryngoscope..

[19] Versius Surgical System (2022) User Manual. VERSIUS®. CMR Surgical Ltd. https://cmrsurgical.com/wp-content/uploads/2022/11/U-00009v15VersiusSurgicalSystem-UserManual-English-UK.pdf. Accessed 04 April 2024.

[20] Brownlee E.M., Slack M. (2022). The role of the versius surgical robotic system in the paediatric population. Children (Basel)..

[21] Alkatout I., Salehiniya H., Allahqoli L. (2022). Assessment of the versius robotic surgical system in minimal access surgery: a systematic review. J Clin Med..

[22] Faulkner J., Arora A., Swords C., Cook E., Rajangam A., Jeannon J.P. (2021). Pre-clinical evaluation of a novel robotic system for transoral robotic surgery. Clin Otolaryngol..

[23] Faulkner J., Arora A., McCulloch P., Robertson S., Rovira A., Ourselin S. (2024). Prospective development study of the Versius Surgical System for use in transoral robotic surgery: an IDEAL stage 1/2a first in human and initial case series experience. Eur Arch Otorhinolaryngol..

